# Analysis of FK506, timcodar (VX-853) and FKBP51 and FKBP52 chaperones in control of glucocorticoid receptor activity and phosphorylation

**DOI:** 10.1002/prp2.76

**Published:** 2014-09-01

**Authors:** Terry D Hinds, Lance A Stechschulte, Fadel Elkhairi, Edwin R Sanchez

**Affiliations:** 1 Center for Diabetes and Endocrine Research, Department of Physiology & Pharmacology, University of Toledo College of MedicineToledo, Ohio, 43614; 2 Center for Hypertension and Personalized Medicine, Department of Physiology & Pharmacology, University of Toledo College of MedicineToledo, Ohio, 43614; 3 Department of Urology, University of Toledo College of MedicineToledo, Ohio, 43614

**Keywords:** Ligands, neuroprotection, nuclear hormone receptors, posttranslational modification, regenerative medicine, steroids

## Abstract

The immunosuppressive ligand FK506 and the FK506-binding protein FKBP52 are stimulatory to glucocorticoid receptor (GR) activity. Here, we explore the underlying mechanism by comparing GR activity and phosphorylation status in response to FK506 and the novel nonimmunosuppressive ligand timcodar (VX-853) and in the presence and absence of FKBP52 and the closely related protein FKBP51. Using mouse embryonic fibroblast cells (MEFs) deficient knockout (KO) in FKBP51 or FKBP52, we show decreased GR activity at endogenous genes in 52KO cells, but increased activity in 51KO cells. In 52KO cells, elevated phosphorylation occurred at inhibitory serine 212 and decreased phosphorylation at the stimulatory S220 residue. In contrast, 51KO cells showed increased GR phosphorylation at the stimulatory residues S220 and S234. In wild-type (WT) MEF cells, timcodar, like FK506, potentiated dexamethasone-induced GR transcriptional activity at two endogenous genes. Using 52KO and 51KO MEF cells, FK506 potentiated GR activity in 51KO cells but could not do so in 52KO cells, suggesting FKBP52 as the major target of FK506 action. Like FK506, timcodar potentiated GR in 51KO cells, but it also increased GR activity in 52KO cells. Knock-down of FKBP51 in the 52KO cells showed that the latter effect of timcodar required FKBP51. Thus, timcodar appears to have a dual specificity for FKBP51 and FKBP52. This work demonstrates phosphorylation as an important mechanism in FKBP control of GR and identifies the first nonimmunosuppressive macrolide capable of targeting GR action.

## Introduction

The macrolide analog FK506 is an immunosuppressive drug that targets FK506-binding proteins (FKBPs). The FKBPs range in molecular weight from 12 to 135 kDa and share a common peptidyl-prolyl isomerase (PPIase) function (MacMillan 2013). Yet, their roles in signaling pathways are as diverse as their size (Harding 2003). FK506 has been shown to suppress the immune system by inhibiting the PPIase function of small molecular weight FKBPs, such as FKBP12. FK506-bound FKBP12 inhibits calcineurin, resulting in increased phosphorylation of the transcription factor NF/AT (nuclear factor of activated T cells), preventing activation of cytokine genes and suppressing the immune system (Clipstone and Crabtree 1992; Snyder and Sabatini 1995). FK506 can also bind the large molecular weight (LMW) immunophilins, FKBP52, and FKBP51, which do not play significant roles in immunosuppression (Yem et al. 1992; Smith et al. 1993a). Instead, FK506-bound LMW FKBPs have important neurotrophic actions, protecting nerves against damage and promoting regeneration (Gold and Villafranca 2003). Importantly, FK506 was shown to be neuroprotective in FKBP12 knockout (KO) mice (Gold et al. 1999) and derivatives of FK506 unable to bind FKBP12 can induce nerve regeneration (Gold 1997; Gold and Villafranca 2003; Gold et al. 2005). These effects have been attributed to FKBP52 (Gold et al. 1999; Price et al. 2005) and underscore the need for macrolide analogs that are selective for LMW FKBPs. One such candidate is timcodar (VX-853), a macrolide analog and non-FKBP12 ligand, whose structure was first reported by Grossman and coworkers (Mullin et al. 2004). Timcodar has been shown to provide neural protection and improves nerve function in several rodent models of experimentally induced neuropathy (Cole et al. 2000; Babine et al. 2005). However, timcodar has not been shown to target the LMW FKBPs.

FKBP51 and FKBP52 are also important regulators of nuclear receptors (Sanchez 2012; Storer et al. 2011). Unlike FKBP12, FKBP51 and FKBP52 contain three tetratricopeptide repeat domains which allow them to bind to the chaperone heat shock protein 90 (Hsp90) and steroid receptor complexes (Owens-Grillo et al. 1996). FKBP52 and FKBP51 were discovered as components of progesterone receptor complexes (Tai et al. 1986; Smith et al. 1993b) and are now known to bind glucocorticoid receptor (GR), androgen, estrogen, and mineralocorticoid receptors (Tai et al. 1986; Sanchez 1990; Bruner et al. 1997). Interestingly, hormone-free GR has a higher affinity for FKBP51 and exchange for FKBP52 occurs upon glucocorticoid binding (Davies et al. 2002, 2005). In addition, FKBP52 is a positive but gene-specific regulator of GR (Wolf et al. 2009), while FKBP51 has an inhibitory effect on GR (Reynolds et al. 1999; Denny et al. 2000). Exposure of cells to FK506 increased (potentiated) the dexamethasone-induced GR response, suggesting that FK506 not only targets FKBP12 but also the larger FKBPs within GR complexes (Ning and Sanchez 1993). Although the mechanism by which FK506 potentiates GR is not fully understood, some progress has been made. Treatment of cell lysates and intact cells with FK506 increase GR hormone-binding affinity (Ning and Sanchez 1995; Davies et al. 2005), a response that can at least partially explain the increase in GR activity, especially at low-hormone concentrations. However, potentiation of GR still occurs at saturating concentrations of hormone, suggesting the existence of additional mechanisms. Interestingly, this effect is not controlled by the PPIase function of FKBP52, as enzymatically dead mutants of FKBP52 can still bind FK506 and potentiate GR (Riggs et al. 2007).

A potential mechanism for FK506 or FKBP control of GR activity is phosphorylation. The N-terminal region of GR contains several serines (S) whose phosphorylation is known to affect GR transcriptional activity (Bodwell et al. 1998; Ismaili and Garabedian 2004; Weigel and Moore 2007). Increased phosphorylation of S203 (murine S212) leads to perinuclear localization and inhibition of human GR. (Wang et al. 2002). Phosphorylation of hS211 (mS220) is acutely upregulated in response to hormone and causes increased nuclear localization and activity (Bodwell et al. 1995; Webster et al. 1997; Wang et al. 2002). Similarly, increased phosphorylation of hS226 (m234) increases GR activity (Webster et al. 1997; Wang et al. 2007). Thus, site-specific phosphorylation of GR represents an important modulatory mechanism. Indeed, we have shown that another GR cochaperone, protein phosphatase 5 (PP5), decreases GR activity by dephosphorylating the receptor at all three serines (Hinds et al. 2011). In this work, we investigated the possibility that FKBP51 and FKBP52 control GR by indirectly affecting its phosphorylation status. In addition, the ability of timcodar to potentiate GR activity is also tested by comparing it to FK506 and determining whether the ligands specifically target FKBP52 or FKBP51 and alter phosphorylation of the receptor.

## Materials and Methods

### Materials

Dexamethasone (Dex), 4-(2-hydroxyethyl)-1-piperazineethanesulfonic acid (HEPES), dulbecco’s modified eagle’s medium (DMEM), powdered medium, Tris, ethylenediaminetetraacetic acid (EDTA), phosphate buffered saline (PBS), sodium orthovanadate, sodium fluoride, protease inhibitor cocktail, dextran coated charcoal, and sodium chloride were all obtained from Sigma (St. Louis, MO). Iron-supplemented bovine calf serum was from Hyclone Laboratories Inc. (Logan, UT). Immobilon-FL polyvinylidenefluoride membrane was obtained from Millipore Corporation (Bedford, MA). Puromycin was from Fisher Scientific (Pittsburgh, PA). FK506 was from Cell Signaling Technology, Inc. (Boston, MA). VX-853 (as timcodar dimesylate) was a gift from Dr. Bruce Gold (Oregon Health and Science University).

### Cell lines and hormone treatment

Mouse embryonic fibroblasts (MEF) were isolated from wild-type (WT), FKBP51 knockout (51KO), and FKBP52 knockout (52KO) E13.5 embryos, as previously described (Yong et al. 2007). Knockdown of FKBP51 in 52KO MEF cells was achieved by lentiviral infection with shRNA (GGTGAAGATATCACTACGAAGAAAGACAG) to mouse FKBP51, as previously described (Hinds et al. 2010). A control 52KO knockdown cell line was made using scrambled shRNA not specific to any known RNA sequence. Cells were routinely cultured in DMEM containing 10% bovine calf serum with 1% penicillin-streptomycin. All of the experiments were carried out on cells at or near confluence with serum that was prestripped of endogenous steroids by 1% (w/v) dextran-coated charcoal. Replicate plates of cells were pretreated for 2 h at 37°C with either FK506, timcodar (VX-853), or DMSO, followed by incubation with ethanol or Dex for an additional 1 h.

### Quantitative real-time PCR analysis

Total RNA was extracted from mouse tissues using 5-Prime PerfectPure RNA Tissue Kit (Fisher Scientific Company, LLC). Total RNA was read on a NanoDrop 2000 spectrophotometer (Thermo Fisher Scientific, Wilmington, DE) and cDNA was synthesized using iScript cDNA Synthesis kit (Bio-Rad, Hercules, CA). PCR amplification of the cDNA was performed by quantitative real-time PCR using qPCR Core kit for SYBR Green I (Applied Biosystems, Carlsbad, CA). The thermocycling glucocorticoid-inducible leucine zipper (GILZ) and serum- and glucocorticoid-regulated kinase (SGK) protocol consisted of 5 min at 95°C, 40 cycles of 15 sec at 95°C, and 30 sec at 60°C and finished with a melting curve ranging from 60 to 95°C to allow distinction of specific products. Primers were designed specific to each gene using Primer Express 3.0 software (Applied Biosystems). Normalization was performed in separate reactions with primers to 18S mRNA (TTCGAACGTCTGCCCTATCAA and ATGGTAGGCACGGCGACTA). All primer sequences were uploaded to the primer database at http://www.primerfinder.com/.

### Whole cell extraction

Cells were washed and collected in 1X PBS followed by centrifugation at 1500*g* for 10 min. The supernatant was discarded and the pellet was resuspended in 1X PBS. After a short spin at 20,800*g* for 5 min at 4°C the pellet was rapidly frozen on dry ice ethanol mix and stored at –80°C for overnight. The frozen pellet was then resuspended in 3 volumes of cold whole cell extract buffer (20 mmol/L HEPES, 25% glycerol, 0.42 mol/L NaCl, 0.2 mmol/L EDTA, pH 7.4) with protease and phosphatase inhibitors (sodium orthovanadate and sodium fluoride) and incubated on ice for 10 min. The samples were centrifuged at 100,000*g* for 5 min at 4°C. Protein levels were measured spectrophotometrically by a Spectra Max Plus spectrophotometer (Molecular Devices Corp., Sunnyvale, CA). The supernatants were used immediately for Western analysis.

### Gel electrophoresis and western blotting

Protein samples were resolved by SDS polyacrylamide gel electrophoresis and electrophoretically transferred to Immobilon-FL membranes. Membranes were blocked at room temperature for 1 h in TBS (10 mmol/L Tris-HCl [pH 7.4], 150 mmol/L NaCl) containing 3% BSA plus phosphatase inhibitors. Incubation with primary antibody was done overnight at 4°C. After three washes in TBST (tris buffered saline plus 0.1% Tween 20), membranes were incubated with infrared anti-rabbit (IRDye 800, green), anti-mouse (IRDye 680, red), or anti-goat (IRDye 800, green) secondary antibodies (LI-COR Biosciences, Lincoln, NE) at 1:15,000 dilution in TBS for 2 h at 4°C. Immunoreactivity was visualized and quantified by infrared scanning in the Odyssey system (LI-COR Biosciences). Antibodies against FKBP51 (sc-11518), FKBP52 (sc-1803), GAPDH (sc-32233), and FiGR (sc-12763) a monoclonal antibody against GR were obtained from Santa Cruz Biotechnologies (Santa Cruz, CA). Phospho-GR S212, S220, and S234 antibodies were made as previously described (Wang et al. 2002) and provided as a gift by Dr. Michael Garabedian (New York University).

### Statistical analysis

Data were analyzed with Prism 5 (GraphPad Software, San Diego, CA) using ANOVA combined with Tukey’s posttest to compare pairs of group means, or unpaired *t* tests. *P* values of 0.05 or smaller were considered statistically significant.

## Results

### FKBP52 and FKBP51 reciprocally regulate GR activity and phosphorylation

**Figure 1:**
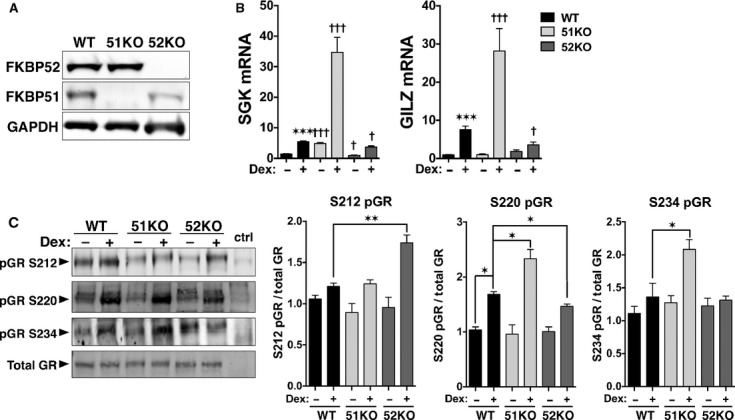
FKBP51 and FKBP52 reciprocally control GR activity and phosphorylation. (A) Western blot analysis of whole cell extracts from WT, FKBP51-KO, and FKBP52-KO MEF cells demonstrating a complete lack of FKBP51 and FKBP52 in the KO cells. GAPDH was used as loading control. (B) qRT-PCR analysis of SGK and GILZ expression in WT, FKBP51-KO, and FKBP52-KO MEF cells following treatment with 100 *μ*mol/L Dex for 2 h. Transcript expression was normalized to 18S mRNA. Data represent the mean ± SEM of three independent experiments, assayed in duplicate. *versus WT control, ^†^KO versus WT. (C) Whole cell extracts of WT, FKBP51-KO and FKBP52-KO MEF cells treated with or without Dex for 1 h were analyzed by Western blotting with antibodies specific to phospho-serines 212, 220, and 234 of mouse GR. FiGR antibody was used to detect total GR. Extracts from COS-7 cells lacking GR were used as negative controls (neg ctrl). Quantitation of GR bands was performed by infrared spectrophotometry. Phospho-GR (pGR) signals were normalized to total GR at each condition. Data represent the mean ± SEM of three independent experiments. Significant differences in transcript expression or protein levels are indicated as follows: *P < 0.05; **P < 0.01; ***P < 0.001. The same parameters apply to † symbol.

FKBP52 and FKBP51 have differential effects on the gene regulatory activities of GR (Denny et al. 2000; Wolf et al. 2009), but the mechanism is unresolved. Here, the mechanism is explored by utilizing MEFs derived from FKBP51 and FKBP52 knock-out mice, 51KO and 52KO MEFs, respectively. The results of Figure 1A show Western blot analysis of each FKBP in the KO cell lines. Although an apparent reduction in FKBP51 is seen in the 52KO cells, quantitation of four independent samples demonstrated no significant reduction (*P* = 0.3359) in the 52KO cells (0.8227 ± 0.1388 SEM) compared to WT cells (1.000 ±0.0973 SEM). Figure 1B shows real-time PCR (qRT-PCR) results measuring GR activity at two endogenous genes, GILZ and SGK. As previously shown (Wolf et al. 2009), 52KO MEFs have significantly reduced Dex-induced GR activity at both genes compared to WT cells. However, 51KO MEFs have increased GR activity at both genes compared to WT MEF cells. Under basal conditions 51KO cells have an increased gene expression of SGK, whereas FKBP52KO cells have a decreased expression. There was no change in basal expression in either cell line for GILZ.

Phosphorylation of GR is known to regulate its transcriptional activity (Bodwell et al. 1998). To determine the effect of FKBP52 and FKBP51 on the phosphorylation of GR, we used antibodies specific to three phospho-serines, S212, S220, and S234 (analogous to human GR S203, S211, and S226), within the transactivation function-1 (TAF-1) domain. WT, 52KO, and 51KO cells were treated with Dex for one hour. Immunoblotting (Fig. 1C) showed no change of S212 phosphorylation in 51KO cells under basal and Dex conditions. However, 52KO cells had a significant increase in Dex-induced S212 phosphorylation. The major Dex-induced site of GR is serine 220 (Wang et al. 2002). Dex treatment of WT cells resulted in an increase in S220 phosphorylation and a significantly higher response in 51KO cells compared to WT. In contrast, 52KO cells had reduced phosphorylation of S220 compared to WT. Treatment of WT or 52KO MEFs with Dex did not enhance phosphorylation at S234. However, there was a significant increase in S234 phosphorylation in 51KO cells.

### Timcodar potentiates GR activity at endogenous genes

**Figure 2:**
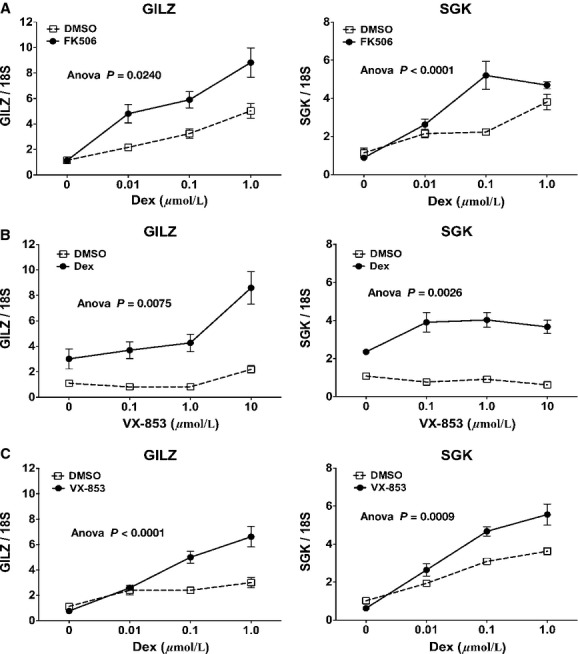
Timcodar and FK506 potentiate GR activity at endogenous genes. (A) qRT-PCR analysis of GILZ and SGK expression in WT MEF cells following treatment with 10 *μ*mol/L FK506 for 2 h followed by 2 h of Dex at the indicated concentrations. (B) qRT-PCR analysis of WT MEF cells treated with the indicated concentrations of timcodar (VX-853) for 2 h followed by 2 h of 1 *μ*mol/L Dex. (C) qRT-PCR analysis of WT MEF cells treated with 10 *μ*mol/L timcodar for 2 h followed by 2 h of Dex at the indicated concentrations. In all cases, transcript expression was normalized to 18S mRNA. Data represent the mean ± SEM of three independent experiments, assayed in duplicate. Statistical analysis by Anova, as indicated.

We have shown that FK506 potentiates Dex-induced GR activity at a minimal MMTV reporter in mouse L929 cells (Ning and Sanchez 1993; Davies et al. 2005). To determine the effect on endogenous genes, we pretreated WT MEF cells with 10 *μ*mol/L FK506 for 2 h, followed by increasing concentrations of Dex and assay for GILZ and SGK expression by qRT-PCR. Treatment with FK506 significantly increased GR-mediated expression of GILZ at all concentrations of Dex and SGK expression at 100 nmol/L Dex (Fig. 2A). To determine if timcodar could similarly potentiate GR, a dose-dependence for timcodar was first performed (0–10 *μ*mol/L) for two hours, followed by 100 nmol/L Dex treatment (Fig. 2B). The timcodar pretreatment significantly potentiated the GR response at both genes. Interestingly, 0.1 *μ*mol/L timcodar potentiated GR activity at SGK but not GILZ, the latter requiring 10 *μ*mol/L timcodar for potentiation. Timcodar treatment alone had no effect on GR activity at either gene. Timcodar at 10 *μ*mol/L was then used to perform a dose-dependence for Dex. The results show that timcodar potentiation begins at 0.1 *μ*mol/L Dex for GILZ and SGK (Fig. 2C) and is even greater at saturating concentrations of Dex (1 *μ*mol/L).

### FKBP52 and FKBP51 differentially regulate potentiation of GR activity by FK506 and Timcodar

**Figure 3:**
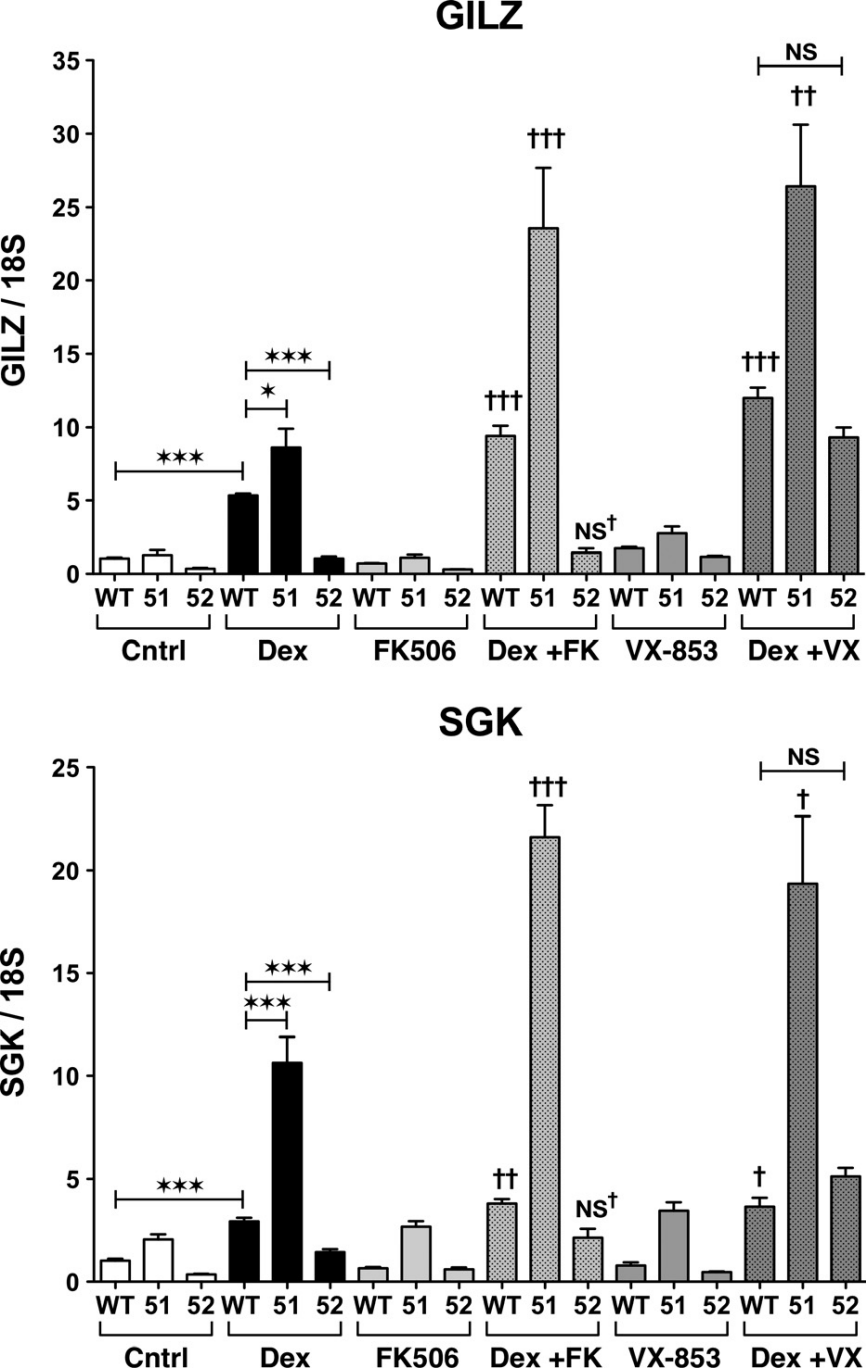
FKBP52 and FKBP51 differentially regulate potentiation of GR activity by FK506 and Timcodar. qRT-PCR analysis of GILZ and SGK in WT, FKBP51-KO, and FKBP52-KO MEF cells treated with 10 *μ*mol/L FK506, 10 *μ*mol/L timcodar (VX-853), or vehicle for 2 h, followed by 2 h of 100 nmol/L Dex or vehicle. GILZ and SGK transcript expression were normalized to 18S mRNA. *versus WT control, ^†^Combination treatments versus their respective Dex alone treatments. Data represent the mean ± SEM of three independent experiments assayed in duplicate. Significant differences in transcript levels are indicated as follows: *P < 0.05; **P < 0.01; ***P < 0.001. The same parameters apply to † symbol.

**Figure 4:**
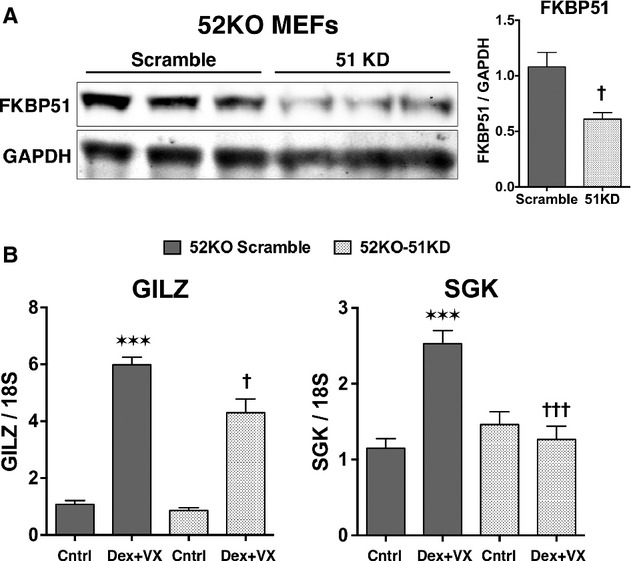
Knock-down of FKBP51 expression prevents Timcodar potentiation of GR activity in FKBP52-KO cells. (A) Western blot analysis of FKBP51 expression in whole cell extracts from FKBP52-KO cells after knock-down of FKBP51 expression using lentiviral delivery of shRNA to FKBP51 (51 KD) or scrambled sequence (Scramble). GAPDH was used as a loading control. Quantitation demonstrates ∼50% reduction in FKBP51 protein following knock-down. Data represent the mean ± SEM of three independent experiments. ^†^KD versus Scramble. (B) qRT-PCR analysis of GILZ and SGK in 52-KO-Scramble and 52-KO-51KD cells treated with 10 *μ*mol/L timcodar (VX-853) or vehicle, followed by 2 h with 100 nmol/L Dex. Transcript expression was normalized to 18S mRNA. Data represent the mean ± SEM of three independent experiments, assayed in duplicate. *versus Scramble control, ^†^KD versus Scramble. Significant differences in transcript levels are indicated as follows: *P < 0.05; **P < 0.01; ***P < 0.001. The same parameters apply to † symbol.

**Figure 5:**
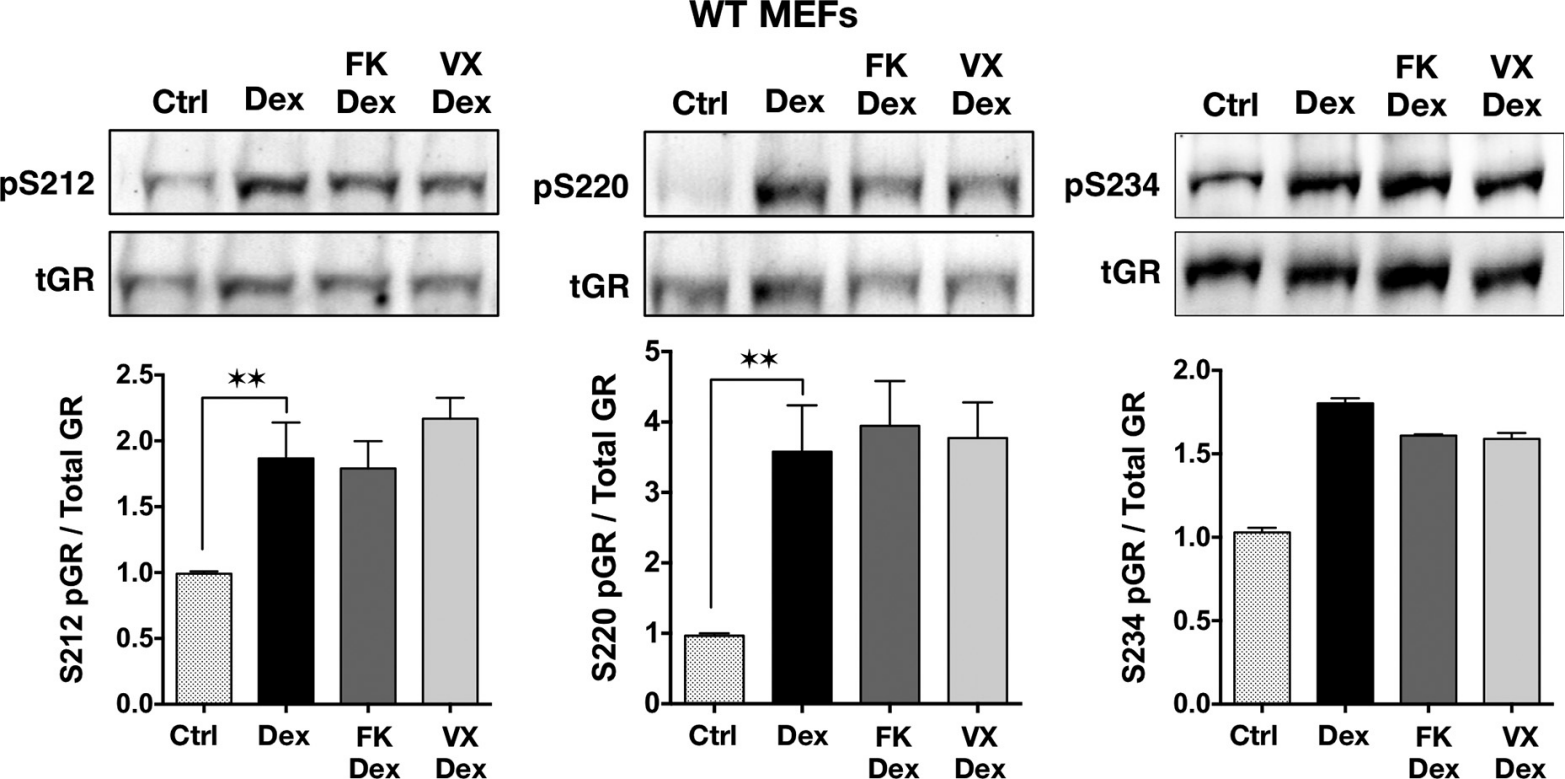
FK506 and Timcodar do not alter Dex-induced phosphorylation of GR. (A) Whole cell extracts of WT MEF cells treated with FK506 (FK), timcodar (VX-853) (VX), or vehicle (Ctrl) for 2 h, followed by 100 nmol/L Dex for 1 h, were analyzed by Western blotting with GR antibodies specific to phospho-serines 212 (p212), 220 (p220), and 234 (p234). FiGR antibody was used to detect the total GR protein (tGR). Quantitation of GR bands was performed by infrared spectrophotometry. Phospho-GR signals were normalized to total GR at each condition. Data represent the mean ± SEM of three independent experiments, assayed in duplicate (p212 and p220), or two independent experiments, assayed in singletons (p234). Significant differences in protein levels are indicated as follows: **P < 0.01.

In all prior studies, the effects of FK506 on GR were performed in WT cells containing FKBP51, FKBP52, and a variety of smaller FKBPs, such as FKBP12. Here, for the first time, we directly assess the contributions of FKBP51 and FKBP52 to both FK506 and timcodar potentiation of GR by using WT, 52KO, and 51KO MEF cells. The cells were pretreated with timcodar or FK506, followed by treatment with Dex and assay for GILZ and SGK expression (Fig. 3). Consistent with the results of Figure 1B, exposure to Dex alone caused increased expression of both GILZ and SGK in 51KO cells compared to Dex-treated WT cells, but reduced expression in 52KO cells. Exposure to FK506 or timcodar alone had no effect on basal levels of either gene in all three cell lines. In response to FK506 plus Dex, expression of both genes was even higher in 51KO cells than in 51KO cells treated with Dex alone, suggesting increased sensitivity to FK506 in the absence of FKBP51. In contrast, 52KO cells treated with FK506 plus Dex showed no increase in gene activity compared to Dex alone, suggesting resistance to FK506 in the absence of this protein. In response to timcodar, a similar increased expression of both genes was seen in 51KO cells compared to 51KO cells treated with Dex alone. However, unlike FK506, timcodar did cause GR activity to increase in 52KO cells to a level commensurate with WT cells treated with Dex and timcodar, suggesting that this drug can rescue GR activity in the absence of FKBP52. To test whether FKBP51 might be the target of the timcodar rescue response, lentiviral delivery of shRNA was used to knock-down expression of FKBP51 in 52KO cells. Approximately 50% reduction in FKBP51 protein was achieved (Fig. 4A). Analysis of Dex-induced GR activity revealed that 51 knock-down in the 52KO cells reduced timcodar potentiation of GILZ and eliminated SGK potentiation (Fig. 4B), suggesting that FKBP51 is the target of timcodar action in those cells. As a last test, the effects of FK506 and timcodar on GR phosphorylation were determined. Using WT cells, Dex-induced phosphorylation at S212, S220, and S234 was once again observed. However, both FK506 and timcodar had little to no effect on Dex-induced phospho-serine levels (Fig. 5).

## Discussion

Prior work has shown that the FKBP FKBP52 and FKBP51 exert differential control of nuclear receptor activity and that the FK506 ligand stimulates (potentiates) the transcriptional activities of some of these receptors, most notably GR. In this work, we have investigated the mechanism by which both of these agents alter GR activity. Using FKBP52 and FKBP51 deficient MEF cells, we show that GR activity at endogenous genes is positively regulated by FKBP52, but inhibited by FKBP51 and that this correlated with altered GR phosphorylation status. In the case of 52KO cells, the decreased GR activity correlated with increased phosphorylation at serine 212 which is known to be inhibitory of GR activity (Wang et al. 2002, 2007), but with decreased phosphorylation at the stimulatory S220 residue (Bodwell et al. 1998; Wang et al. 2002). In contrast, 51KO cells showed no change in phosphorylation of S212, but did show increased phosphorylation at two stimulatory GR residues S220 and S234 (Wang et al. 2007). The capacity of each FKBP to alter GR phosphorylation is intriguing, as neither protein has intrinsic phospho-regulatory activity. Thus, they must be achieving this indirectly. We speculate that FKBP51 does this through its recently published ability to chaperone the Akt-specific phosphatase PHLPP (Pei et al. 2009; Fabian et al. 2013). Loss of FKBP51 might, therefore, result in elevated Akt activation leading to downstream events targeting GR at serines 220 and 234. Of course, FKBP51 might regulate other kinase cascades. FKBP52 has also been shown to regulate phosphorylation mechanisms, including the Ras/Raf/Mapk pathway (Gold and Villafranca 2003; Price et al. 2005) and regulation of the Src tyrosine kinase Lck (Scroggins et al. 2003). Future studies will be needed to identify and characterize the specific phosphorylation mechanisms controlled by each FKBP and its impact on GR.

In this work, we also demonstrate that the nonimmunosuppressive macrolide timcodar (VX-853), like FK506, can potentiate GR transcriptional activity. Using 52KO and 51KO MEF cells, we show that the major target of FK506 action on GR is FKBP52, rather than FKBP51, as FK506 potentiated GR activity in 51KO cells but could not do so in 52KO cells. Like FK506, timcodar potentiated GR in 51KO cells, but it also increased GR activity in 52KO cells and this effect required FKBP51. These data suggest that timcodar may have dual specificity for each FKBP. Importantly, these results also suggest that the purported ability of timcodar to act as an inhibitor of multidrug resistance (MDR) proteins (Mullin et al. 2004; Mitchell et al. 2005) is probably not the basis for its GR potentiation effect, because increased intracellular retention of Dex due to timcodar inhibition of MDR would presumably occur in the 52KO-51KD cells. Yet, timcodar had little to no effect on GR activity in these cells. Instead, it appears that timcodar may be controlling GR principally by targeting the LMW FKBPs. We tested the intriguing possibility that FK506 and/or timcodar targeting of the LMW FKBPs altered GR by affecting phosphorylation. Yet, no effects at serines 212, 220, or 234 were seen with either ligand. Thus, it appears that unliganded FKBP51 and FKBP52 can affect phosphorylation pathways in a way that is unaffected by FK506 or timcodar. Although it is possible that FK506 or timcodar are targeting other phospho-residues on the GR, our results are consistent with several published facts. First, Smith and colleagues have shown that FKBP52 stimulation of GR is completely independent of its PPIase activity, as enzymatically null mutants still potentiated GR activity (Riggs et al. 2007). Thus, the PPIase function of LMW FKBPs may also be inconsequential to regulation of phosphorylation cascades. Second, we have shown that FK506 increases GR hormone-binding affinity (Ning and Sanchez 1995; Davies et al. 2005), yet loss of FKBP52 had no effect on this function (Wolf et al. 2009), further indicating that FK506 binding to FKBP52 is a gain-of-function phenomenon when it comes to control of the hormone-binding function. Taken as a whole, these observations suggest that FKBPs and their cognate ligands can exert a multifactorial control over GR transcriptional activity through FKBP-dependent regulation of phosphorylation and FK506-dependent regulation of the hormone-binding event.

This is the first study to demonstrate that timcodar can potentiate GR activity, similar to FK506. However, unlike FK506, the timcodar mechanism cannot involve the FKBP12/calcineurin pathway responsible for immunosuppression. Indeed, in this work we also show that GR potentiation by FK506 is independent of FKBP12/calcineurin, as FK506 stimulation of GR was lost in FKBP52-KO cells. Although several non-FKBP12 ligands have been shown to be useful in neuroregeneration, without the immunosuppression that occurs with FK506 (Gold et al. 1999), drugs that increase nerve regeneration by specifically targeting the LMW FKBPs have not been developed. Timcodar can now be viewed as such a candidate and may serve as the basis for drug design targeting the neuroprotective properties of LMW FKBPs. It is interesting to note that timcodar dimesylate has already been shown to be an orally bioavailable ligand protective against experimentally induced neuropathies in several rodent models, such as pyridoxine-induced nerve damage and streptozotocin-induced type 1 diabetes (Cole et al. 2000). However, timcodar dimesylate has not been effective in two preclinical human trials utilizing capsaicin-induced nerve injury (Polydefkis et al. 2006) or intracutaneous axotomy (Hahn et al. 2006). This discrepancy may be attributable to many factors, such as use of healthy individuals in the human trials, the nature of the neurotrauma, or an inability of timcodar to target human FKBP cognates. Indeed, Hausch and colleagues have shown that timcodar does not bind purified FK1 (PPIase domain) fragments of human FKBP51 or FKBP52 (Gopalakrishnan et al. 2012), suggesting that timcodar is inactive in human FKBPs, or that it targets other regions of the proteins, such as the FK2/PPIase-like domain. Further and more complete in vitro binding studies using mouse LMW FKBPs, as well as the development of timcodar derivatives will be necessary to address these issues.

Another potential benefit of our study is reflected in the ability of timcodar to enhance glucocorticoid signaling in the absence of FKBP52 by targeting FKBP51. Timcodar may, therefore, be a potential therapy for disease states in which FKBP52 is suppressed or absent. Our laboratory and others have shown that KO of FKBP52 in mice results in several pathological conditions, such as altered signaling of androgen receptor leading to hypospadias and reduced male fertility (Cheung-Flynn et al. 2005; Yong et al. 2007), reduced progesterone receptor activity leading to defective uterine implantation (Tranguch et al. 2005; Yang et al. 2006), and reduced GR activity leading to increased susceptibility to diet-induced steatosis (Warrier et al. 2010). Finally, a study by Manabe et al. (2002) demonstrated that FKBP52, but not FKBP12, was downregulated in the pathogenesis of early-stage amyotrophic lateral sclerosis.

In summary, these studies identify an important new mechanism by which FKBP51 and FKBP52 regulate steroid receptor action, namely, phosphorylation. Although this mechanism is likely to be indirect (through regulation of phosphorylation cascades), its further investigation may uncover the basis for FKBP reciprocal modulation of important glucocorticoid-mediated cellular responses, such as apoptosis, immunosuppression, and control of lipid and carbohydrate metabolism. Indeed, our other finding in this work that timcodar, like FK506, can potentiate GR activity, suggests that these compounds may be useful in combination therapy with glucocorticoids for the purposes of immunosuppression, such as in organ transplantation, or for treatment of inflammatory conditions. At the very least, FK506 and/or timcodar may allow for lower effective glucocorticoid dosing, thus helping to ameliorate the steroid’s many side-effects.

## Abbreviations

FKBP: FK506-binding protein
GR: glucocorticoid receptor
PPIase: peptidyl-prolyl cis-trans isomerase
TPR: tetratricopeptide repeat

## Supporting Information

**Figure S1.** Comparison of FK506 and VX-853 chemical structures.

**Figure S2.** Demonstration of no significant change in FKBP51 expression in 52KO MEF cells. These data show Western blots of FKBP51 from three independent experiments in which rescue (ResQ) expression of Flag-tagged FKBP51 and Flag-tagged FKBP52 was being tested in each cell line. Normal (unrescued) WT, 51KO, and 52KO MEFs cells were used as controls. Blots of Flag-tagged proteins not shown, as rescue expressions were not successful and were not relevant to the manuscript. Quantitation of FKBP51 from these blots (WT, 51KO, and 52KO lanes only) and from the blot of Figure 1A were compiled and reported in the text under Results.
