# Supplementary figures and images for: Analysis of FK506, timcodar (VX-853) and FKBP51 and FKBP52 chaperones in control of glucocorticoid receptor activity and phosphorylation
Source: Pharmacol Res Perspect. 2014 Sep 1;2(6):e00076. doi: 10.1002/prp2.76 (PMC4186452; doi:10.1002/prp2.76)

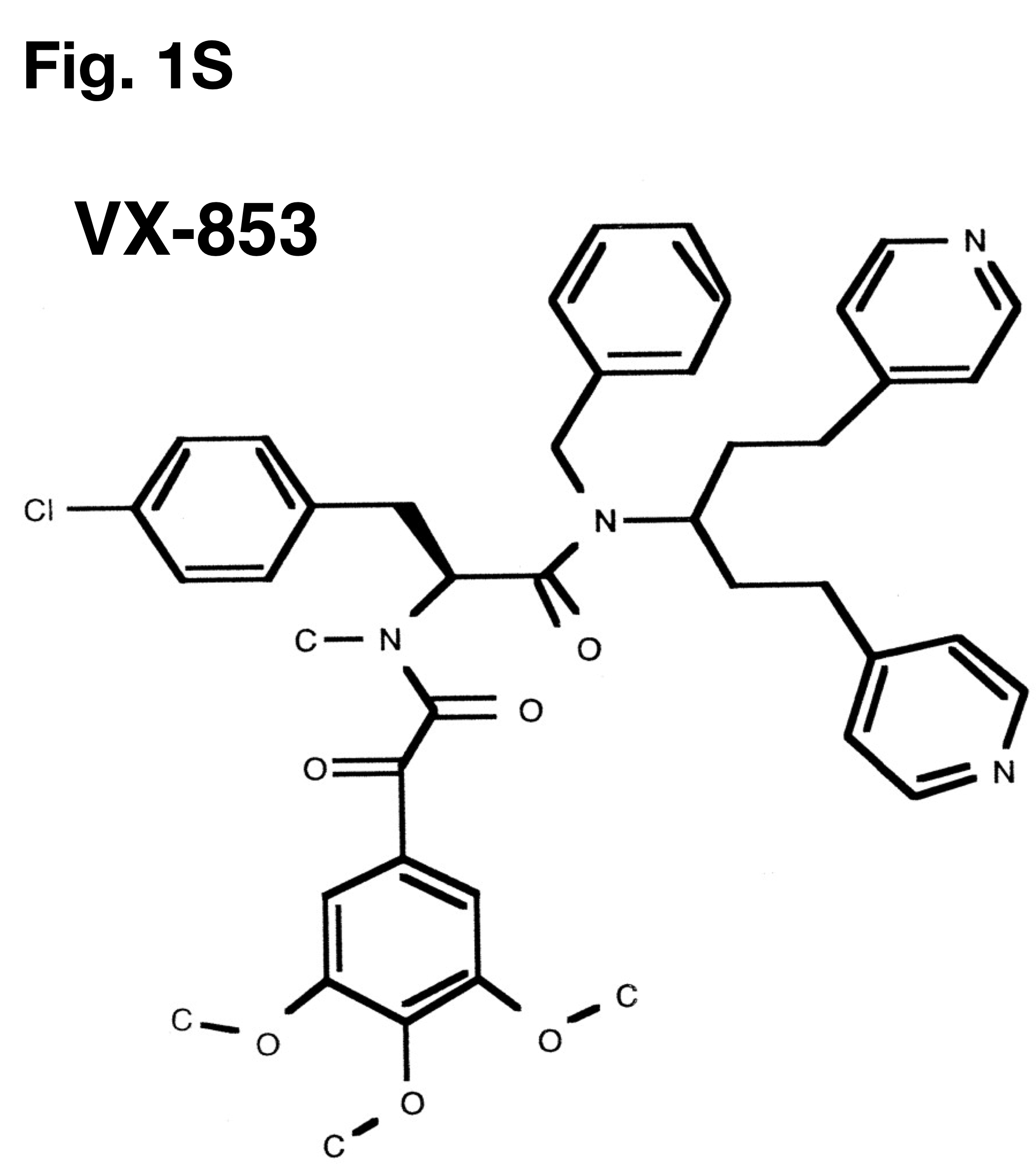

Supplement: Supplementary file 1 — Figure S1. Comparison of FK506 and VX-853 chemical structures. [file prp20002-e00076-SD1.tiff]

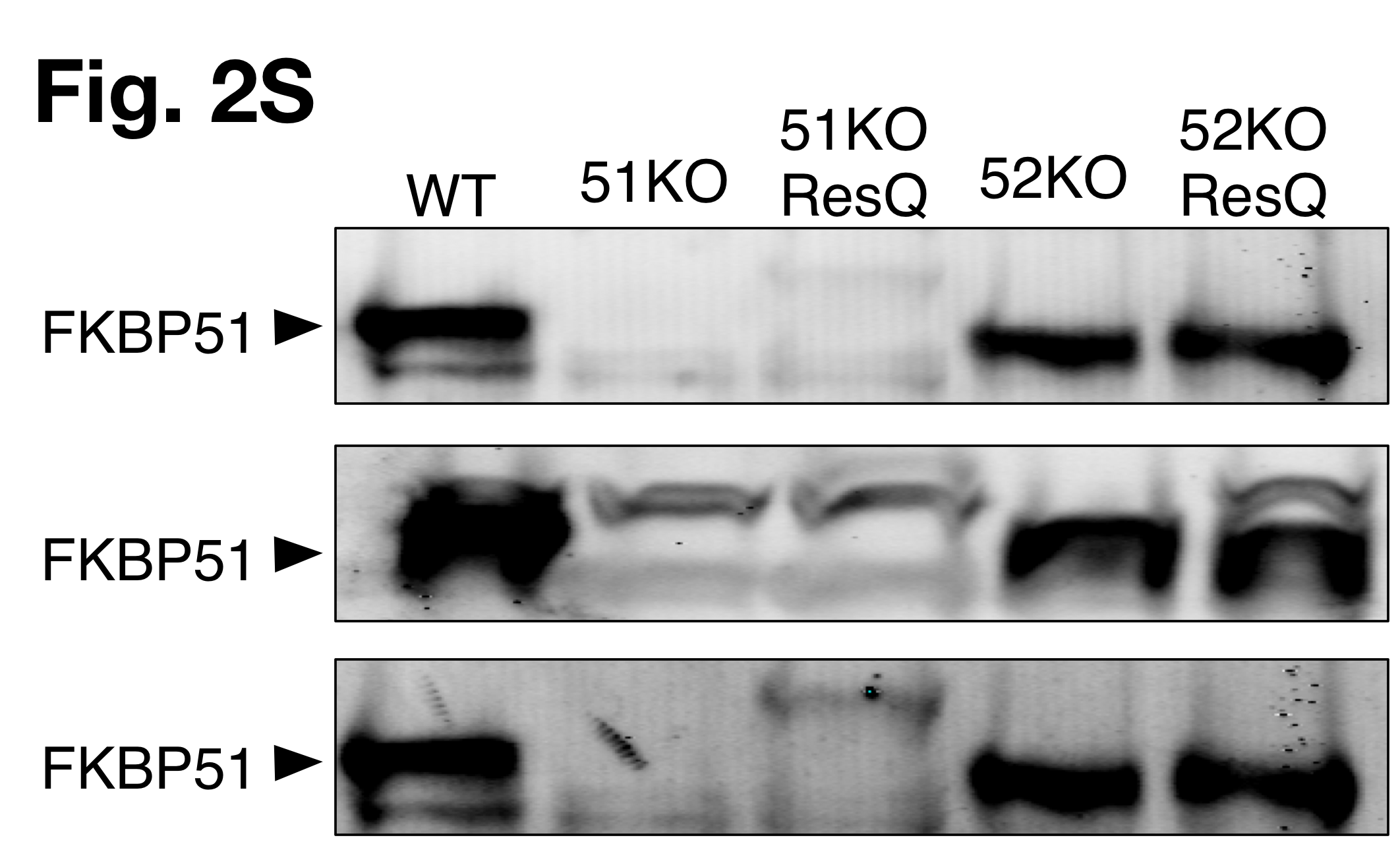

Supplement: Supplementary file 2 — Figure S2. Demonstration of no significant change in FKBP51 expression in 52KO MEF cells. These data show Western blots of FKBP51 from three independent experiments in which rescue (ResQ) expression of Flag-tagged FKBP51 and Flag-tagged FKBP52 was being tested in each cell line. Normal (unrescued) WT, 51KO, and 52KO MEFs cells were used as controls. Blots of Flag-tagged proteins not shown, as rescue expressions were not successful and were not relevant to the manuscript. Quantitation of FKBP51 from these blots (WT, 51KO, and 52KO lanes only) and from the blot of Figure 1A were compiled and reported in the text under Results. [file prp20002-e00076-SD2.tiff]
