# Supplementary material for: Analysis of FK506, timcodar (VX-853) and FKBP51 and FKBP52 chaperones in control of glucocorticoid receptor activity and phosphorylation
Source: Pharmacol Res Perspect. 2014 Sep 1;2(6):e00076. doi: 10.1002/prp2.76 (PMC4186452; doi:10.1002/prp2.76)
Supplement: Supplementary file 3 [file prp20002-e00076-SD3.doc]

**Supplemental Figure Legends**

**Fig. 1S.** Comparison of FK506 and VX-853 chemical structures.

**Fig. 2S.** Demonstration of no significant change in FKBP51 expression in 52KO MEF cells. These data show Western-blots of FKBP51 from three independent experiments in which rescue (ResQ) expression of Flag-tagged FKBP51 and Flag-tagged FKBP52 was being tested in each cell line. Normal (un-rescued) WT, 51KO and 52KO MEFs cells were used as controls. Blots of Flag-tagged proteins not shown, as rescue expressions were not successful and were not relevant to the manuscript. Quantitation of FKBP51 from these blots (WT, 51KO and 52KO lanes only) and from the blot of Fig. 1A were compiled and reported in the text under Results.
